# Development and implementation of a MediaPipe-based AI teaching-learning model in school physical education for health promotion

**DOI:** 10.3389/fpubh.2026.1786427

**Published:** 2026-03-20

**Authors:** Donghyun Kim, Yongchul Kwon, Gunsang Cho, Minseo Kang

**Affiliations:** Department of Physical Education, Pusan National University, Busan, Republic of Korea

**Keywords:** artificial intelligence, computational thinking, Mediapipe, participatory action research, physical education, real-time pose estimation, school health promotion

## Abstract

**Introduction:**

Artificial intelligence (AI) technologies are increasingly used in school physical education (PE) to provide real-time feedback and support instruction that promotes youth physical activity and health. However, many AI applications remain top-down and expert-driven, focusing on technical validation in controlled settings and paying little attention to everyday school contexts or the needs of lower-fitness students. This study aimed to develop and implement a MediaPipe-based AI teaching–learning program for health-oriented middle school PE.

**Methods:**

A participatory action research (PAR) design with rapid prototyping was conducted over three months in one public middle school. PE teachers and 9th-grade students participated as co-researchers and co-developers. A web-based program using MediaPipe Pose was iteratively designed to recognize selected fitness movements and provide immediate visual and auditory feedback, with QR-code access and automatic logging. Data from semi-structured group interviews, observations, teachers' reflective journals, and student-created artifacts were analyzed using thematic analysis.

**Results:**

Across three PAR cycles, the prototype evolved from a simple elbow-angle counter into a system that incorporated body alignment, tracking for isometric exercises, multimodal feedback, and automatic data recording. Teachers used the program to design lesson-specific recognition rules, monitor students' exercise participation, and support individual growth. Students deepened their understanding of exercise principles and engaged in computational thinking while experimenting with movements and refining feedback conditions.

**Conclusions:**

A participatory, school-based approach enabled MediaPipe-based pose estimation to be reconfigured into a pedagogically meaningful, health-oriented program for middle school PE, suggesting that AI-supported PE can contribute to more inclusive, data-supported school health promotion.

## 1 Introduction

In the era of digital transformation, artificial intelligence (AI) technologies are rapidly reshaping educational and health practices by enabling personalized feedback, real-time learning support, and data-informed decision-making (1, 2). This trend is increasingly evident in school physical education (PE), which has been recognized as a critical setting for promoting youth physical activity, preventing lifestyle-related diseases, and reducing health disparities among children and adolescents (3, 4). Within this context, AI-based approaches such as motion recognition, posture correction, and performance analysis have been introduced as potential means to enhance the quality and equity of PE instruction while contributing to broader public health goals (5).

In PE, observing learners' movements and providing timely corrective feedback constitute core teaching and learning processes that are directly linked to students' opportunities to develop fundamental movement skills and maintain healthy levels of physical activity (6). However, in authentic classroom settings, teachers are required to supervise and instruct many students simultaneously, which constrains their ability to monitor individual performances in detail and to deliver immediate, personalized feedback, especially for less active or lower-fitness students (6, 7). Against this backdrop, computer-vision-based pose estimation technologies have gained attention for their capacity to extract body-joint information in real time and quantitatively analyze movement patterns, thereby supporting observation, evaluation, and feedback processes in PE lessons and potentially strengthening the health-promoting function of school PE (8–11).

At the same time, current applications of AI in PE and health-related education are often characterized by a top-down approach in which teachers adopt pre-designed systems developed by technical experts (12). Such approaches frequently fail to account for pedagogical context, classroom constraints, and learners' developmental and health needs, increasing the risk that AI is used in a superficial, technology-driven manner that does little to advance health equity or meaningful engagement in physical activity (13, 14). From an educational and public health perspective, what matters is not the technology itself but how it is meaningfully integrated into specific teaching and learning contexts to support safe, inclusive, and health-oriented participation (1, 13). This implies that teachers need to move beyond the role of passive technology users and instead act as pedagogical designers who can adapt, reinterpret, and transform AI technologies in accordance with instructional goals, learner characteristics, and local health priorities (15, 16).

In this study, when we describe existing AI applications in PE as “top-down,” we refer not to the underlying algorithmic architecture but to the ways these systems are pedagogically adopted in schools. More specifically, we use “top-down” to describe closed, pre-packaged applications in which recognition rules and feedback mappings are fixed by developers and cannot be readily modified by teachers or learners *in situ*. Pose estimation frameworks such as MediaPipe inevitably rely on expert-defined models and pre-trained parameters; this reflects technological agency, that is, model-level constraints embedded by technical experts. By contrast, our focus is on pedagogical agency, that is, how teachers and students configure, reinterpret, and repurpose these models for specific instructional goals in PE. Our contribution is not to propose a “non-expert” AI. Rather, we show how teachers and students can exercise pedagogical agency on top of an expert-driven pose estimation backbone by iteratively authoring and revising rule-based recognition criteria and feedback logic within authentic lessons. This framing helps reconcile the technical necessity of pre-trained models with the bottom-up, student-centered goals of participatory design and aligns with recent discussions on human-centered AI integration that supports learners' and teachers' active roles in physical skill learning and PE contexts (17–20).

Within AI-based educational research, code-free machine learning tools such as Teachable Machine have been widely used to introduce basic AI concepts and stimulate learner interest, but they are limited in their capacity to provide fine-grained, biomechanically meaningful feedback for health-related movement learning (21–23). In contrast, pose estimation frameworks such as MediaPipe enable real-time tracking of multiple body joints using standard cameras, allowing for the calculation of joint angles, alignments, and temporal changes in movement that can underpin more specific and personalized feedback aligned with PE and health promotion objectives (8, 24). Recent studies suggest that gamified intelligent tutoring and AI-facilitated peer-assisted learning can enhance students' motor skill performance, motivation, and basic psychological needs satisfaction in somatic practice and physical skill learning contexts (17, 19), while AI-supported auto-assessment and feedback approaches can promote reflective practice and self-regulated learning in PE and related physical activity settings (18, 20). However, many MediaPipe- and OpenPose-based applications have predominantly been designed and validated as expert-developed “virtual fitness coach” or intelligent tutoring systems, with limited attention to the complexities of school PE curricula or to the pedagogical roles of teachers and students in co-configuring AI-supported, health-oriented practice.

Against this background, the present study adopts participatory action research (PAR) to investigate the collaborative development and classroom implementation of a MediaPipe-based AI teaching–learning model for health promotion in middle school PE (7, 25). By involving PE teachers and students as co-researchers and co-developers, the study seeks to explore how AI technology can be reconfigured in ways that are pedagogically meaningful, context-responsive, and sustainable, while also enhancing opportunities for students, particularly those with lower fitness or confidence, to engage in safe, data-supported physical activity (3, 26).

Specifically, this study addresses the following research questions:

- Through what processes is a MediaPipe-based AI teaching–learning program collaboratively developed in the context of middle school PE for health promotion?
- What pedagogical and health-related implications emerge when the developed program is implemented in authentic middle school PE classes?

## 2 Research design and methods

### 2.1 Research design and context: participatory action research with rapid prototyping

This study employed participatory action research (PAR) to develop and implement a MediaPipe-based AI teaching–learning model in middle school physical education (PE). PAR was selected because it positions practicing teachers as active agents who identify instructional problems within their own contexts and collaboratively generate context-responsive solutions (25). Through this approach, the study sought to move beyond top-down technology adoption and instead enable teachers and students to participate as co-designers, ensuring that the resulting AI model would be educationally meaningful and sustainable in practice.

To support iterative technological development within the PAR cycles, the rapid prototyping instructional system design (RPISD) model was incorporated as a complementary framework. RPISD emphasizes rapid, iterative development and the immediate incorporation of user feedback, which aligns closely with the collaborative and reflective principles of PAR (27, 28). In this integrated design, PAR guided the educational inquiry, while rapid prototyping facilitated the flexible refinement of the AI tool through continuous interaction with classroom practice.

The study was conducted over approximately three months (August–October 2025) at a public middle school in Ulsan, South Korea. The AI model was implemented during regular 9th-grade PE lessons within a physical fitness unit in the “health” area of the curriculum. PE teachers and students from the same school collaboratively developed and used the AI teaching–learning model in their everyday lesson context, enabling close interaction, repeated feedback, and the effective operation of the cyclical PAR–RPISD design in authentic PE classes.

Importantly, in this integrated design, the PAR–RPISD process not only determined how the AI tool was used pedagogically, but also shaped the core recognition and feedback logic of the program, as teachers and students repeatedly adjusted the rule sets that translated pose-estimation data into health-oriented cues.

### 2.2 Research tools: Mediapipe-based program development environment

In this study, Google's open-source framework MediaPipe was adopted as the core technology for developing the AI teaching–learning program. MediaPipe provides the capability to track 33 body pose landmarks in real time within a web browser without the need for separate server configuration (29). Because it runs using a standard webcam and is not dependent on specific hardware or operating systems, it was considered advantageous for ensuring accessibility and scalability in school settings that use diverse devices. To enhance accessibility in classroom settings, the program was deployed as a web-based application accessible via a QR code. Students scanned the QR code using their school-provided tablets, which immediately opened the program in a web browser without requiring installation, login, or additional configuration. This design minimized procedural barriers and supported seamless integration into routine PE lessons. The system was implemented in a 1:1 device environment using school-issued tablets mounted on portable stands, so that neither teachers nor students were required to hold the devices manually during use.

Based on this technical consideration, the prototype was implemented using JavaScript and the p5.js library, to support compatibility with the various devices used by students. Within this development environment, the prototype was iteratively refined in alignment with the cyclical PAR process described in Section 3.4, incorporating participant feedback immediately and progressively improving the program.

Beyond adopting MediaPipe as a pose estimation backbone, we implemented the AI program as a rule-based environment rather than a fixed, pre-packaged application. Movement recognition and feedback were structured as editable rules that connected subsets of the 33 pose landmarks to biomechanically meaningful conditions (e.g., joint-angle ranges, alignment of body segments, temporal thresholds). These rules were represented in a parameterized format (e.g., configurable landmark pairs, angle thresholds, repetition counts, and feedback messages) so that they could be modified without changing the underlying pose estimation model.

Within the PAR–RPISD cycles, the teacher–student co-researcher group iteratively authored and revised these rules in response to observed classroom issues. For example, an initial push-up rule based primarily on elbow flexion was expanded to include shoulder–hip–knee alignment and minimum range-of-motion criteria, and a plank rule was redesigned from a simple “hold” condition to a time-based tracking rule with continuous alignment checks. In this way, the system functioned not as a single pre-designed “virtual coach” but as a flexible, co-configured rule set built on top of an expert-defined pose estimation backbone. To avoid compromising the technical validity of the feedback, the editable parameters were constrained to biomechanically meaningful ranges derived from the pose-estimation model and the teachers' domain expertise.

### 2.3 Participants

This study employed a collaborative and participatory approach in which teachers and students jointly engaged in the development of the AI teaching–learning model. Participants were selected through purposive sampling to recruit information-rich cases relevant to the research questions (30).

The teacher participants consisted of five physical education teachers at the participating school (average teaching experience: 8 years, range: 5–15 years). They were selected based on their strong interest in AI integration and instructional expertise. Throughout the study, the teachers participated as co-action researchers, taking leading roles in identifying instructional problems, generating design ideas, implementing prototypes in lessons, and engaging in collaborative reflection.

The student participants were five 9th-grade students (three boys and two girls) drawn from the teachers' classes. Selection criteria included interest in physical education and AI technologies, and prior experience with coding or related digital tools. Students participated as co-designers and key informants, contributing to the conceptualization and iterative improvement of the AI model alongside the teachers.

The researcher did not take the lead in direct lesson design but instead acted as a facilitator and critical friend. Specifically, the researcher provided methodological support to help the teacher–student group operate the PAR cycles smoothly, organized workshops and meetings, and managed the collected data systematically. In addition, the researcher posed critical questions during regular discussions to help teacher participants reflect more deeply on their own practices.

### 2.4 Research procedure

This study was conducted over approximately three months, from August to October 2025, through one preparatory phase and three main action cycles. Each cycle followed four iterative stages: planning → Acting and Developing → Observing → Collaborative Reflecting. The core problems, key activities, and main reflections for each cycle are summarized in Table 1.

**Table 1:** Summary of PAR cycles: iterative development of the AI model.

**Cycle**	**Key problem (plan)**	**Core action (act & develop)**	**Key reflection (observe & reflect)**
Cycle 1	Inaccurate rep counting	Prototype v1.0: elbow angle only	Algorithm flawed; Body alignment is crucial.
Cycle 2	Low engagement; Limited feedback	Prototype v2.0: body alignment + auditory feedback	Accuracy improved; Need for time-based exercise tracking.
Cycle 3	Lack of data logging & exercise variety	Prototype v3.0: timer for isometric exercise + data logging	Utility confirmed; revealed potential for AI-integrated PE lessons.

In the preparatory phase (August), the teacher and student participants engaged in a joint workshop to diagnose major issues in current physical education classes and to agree on a shared research goal: developing an AI program capable of real-time posture correction and automatic recording.

The three action cycles proceeded as summarized in Table 1, with each cycle identifying a focal problem, implementing a corresponding prototype, and generating directions for refinement through observation and collaborative reflection. Following the completion of the cycles, a concluding reflection workshop was conducted to examine the educational implications and overall meanings of the final model and the research process.

### 2.5 Data collection

To capture participants' experiences and interactions during the participatory action research (PAR) process, data were collected from multiple sources to ensure qualitative triangulation. First, semi-structured group interviews (approximately 40 min each) were conducted with teacher and student participants at the end of each PAR cycle. The interviews focused on experiences with the AI model, perceived changes, and suggestions for improvement, and were audio-recorded with participants' consent and transcribed verbatim.

Second, participant observation was conducted, with the researcher attending weekly physical education classes and participant meetings as a facilitator and recording field notes on in-class interactions, patterns of AI model use, and emergent issues. Third, reflective materials and artifacts were collected, including teachers' reflective journals written after each cycle and student-generated artifacts such as sketches from idea-generation meetings and written reflections on using the program.

### 2.6 Data analysis

All qualitative data (including interview transcripts, observation field notes, reflective journals, and student-generated artifacts) were analyzed using thematic analysis following Braun and Clarke (31). The first author conducted iterative readings of the dataset and performed initial open coding to identify meaningful units related to the research questions. Codes were refined and organized into higher-order thematic categories through constant comparison and analytic memo writing. To enhance analytic rigor and minimize potential interpretive bias, a second researcher independently coded a randomly selected 25% of the dataset. Inter-rater reliability was assessed using percent agreement and Cohen's Kappa coefficient. The overall percent agreement was 85%, and Cohen's Kappa was κ = 0.82, indicating almost perfect agreement. Discrepancies were discussed until consensus was reached, and the codebook was refined accordingly. No CAQDAS software was used; coding was conducted systematically using structured coding sheets.

### 2.7 Operational technical performance

As this study focused on educational implementation rather than algorithm development, the underlying MediaPipe Pose model was not retrained or benchmarked against controlled ground-truth datasets. The primary aim was to examine how a rule-based movement feedback system could be configured and utilized within authentic physical education settings.

To ensure operational transparency, real-time processing performance was measured using browser-based monitoring on the tablets employed during classroom instruction. Under typical instructional conditions, the system operated at an average of 25.5 frames per second (FPS), supporting real-time feedback without perceptible delay during student movement execution.

In addition, operational stability was examined during authentic classroom use. Landmark tracking remained stable across regular instructional activities, and no systematic recognition breakdown was observed during standard movement tasks. These observations indicate that the system functioned reliably within the intended educational environment.

### 2.8 Ensuring rigor and trustworthiness of the study

To ensure the rigor and trustworthiness of this qualitative study, the research process was guided by Lincoln and Guba's (32) criteria of credibility, transferability, dependability, and confirmability. Credibility and confirmability were enhanced through data triangulation, member checking, peer debriefing, and the maintenance of a reflexive journal. Transferability and dependability were addressed by providing thick descriptions of the research context and systematically documenting key methodological decisions to establish an audit trail. In addition, the study received ethical approval from the Institutional Review Board of Pusan National University (PNU IRB/2025_135_HR), and written informed consent was obtained from all participants and their legal guardians, ensuring that the research process was conducted in an ethically responsible manner.

## 3 Results

### 3.1 Development of mediapipe-based AI teaching-learning program

#### 3.1.1 Structure and characteristics of the initial prototype implemented through participatory design

The AI-supported teaching–learning program developed in this study was designed through a participatory process that directly reflected the educational needs and classroom experiences of physical education teachers and students, with a particular focus on promoting health within school PE lessons. Rather than introducing a pre-designed technological solution, the program emerged through iterative dialogue and experimentation within actual middle school PE classes. From the outset, accessibility, pedagogical usability, and contribution to students' health-related exercise participation were identified as core design principles.

To ensure practical feasibility in school settings, the program was implemented as a web-based application that required no separate installation and could be used across diverse devices. This design choice was jointly determined by teacher and student participants to minimize technical barriers and allow seamless integration into regular PE lessons. As a result, the program could be readily accessed during class using standard devices and supported real-time use without disrupting existing instructional routines.

**Figure 1:**
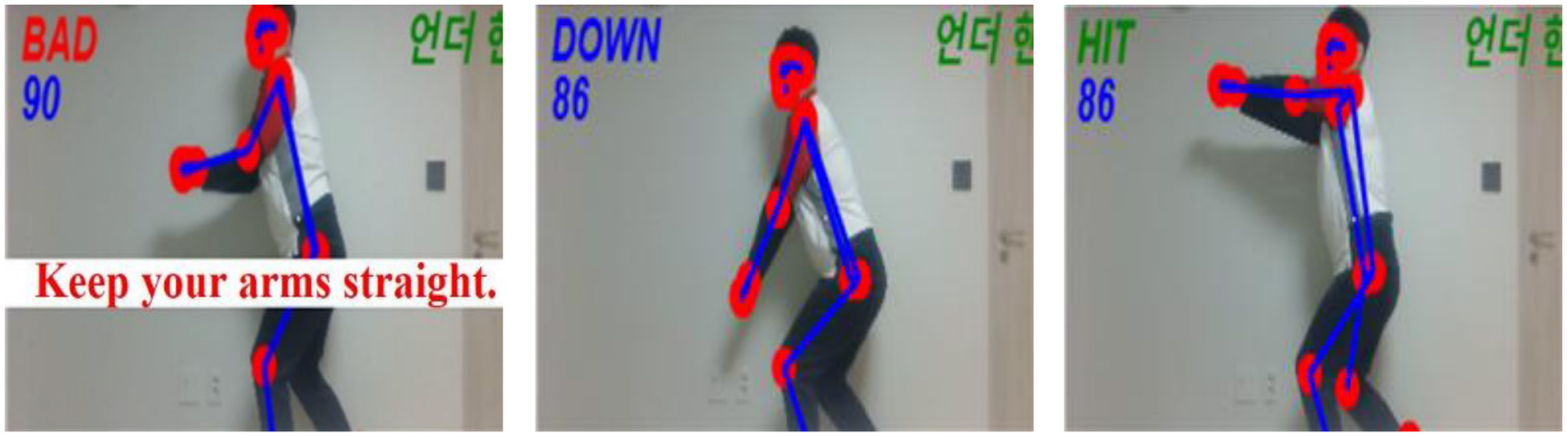
AI-based motion recognition of the underhand pass: from an incorrect “BAD” posture with bent arms through the “DOWN” phase to the “HIT” phase with correct arm extension. The image displays a single participant for privacy protection and interface clarity. The system was implemented during regular, full-class PE sessions in a typical school environment.

A central feature of the program was its capacity to provide immediate and visually intuitive feedback on students' movement performance. As illustrated in Figure 1, students' movements were recognized in real time and categorized into qualitatively meaningful states such as preparation, execution, and incorrect posture. This enabled students to monitor their own performance while performing physical tasks. Teacher participants emphasized that this feature directly addressed a persistent instructional challenge in large PE classes, namely the difficulty of observing and correcting individual students' movements simultaneously.

Through successive participatory action research cycles, the feedback functions were refined based on classroom observations and participant input. In response to students' requests for more motivating and self-monitoring-oriented features, a repetition-counting function was incorporated to allow students to immediately confirm their performance outcomes during practice. Teachers reported that this function increased students' engagement and provided concrete performance information that supported individualized feedback and post-activity reflection.

Across the participatory action research cycles, the program evolved from a basic motion-recognition tool into an AI-supported feedback system aligned with the pedagogical goals and constraints of school physical education. The iterative refinement process prioritized educational relevance, immediacy of feedback, and ease of classroom use rather than technical precision alone. Consequently, the final version of the program functioned as a practical instructional aid that supported students' active engagement and enabled teachers to extend their feedback capacity within the dynamic context of PE lessons. For transparency, a detailed overview of the system's operational workflow is provided in Appendix A.

#### 3.1.2 Support for teaching and learning through improvements in core functions

In this study, the AI-supported program was iteratively refined to address instructional challenges identified during classroom use and to better support teaching and learning in physical education lessons. Rather than focusing on technical optimization alone, functional improvements were guided by observations of student performance and feedback from teacher and student participants during the participatory action research cycles.

One key area of refinement involved improving the reliability of movement recognition in authentic classroom conditions. During early testing, teachers observed that certain movements were occasionally misclassified, which limited the usefulness of automated feedback during practice. In response, the recognition criteria were expanded to enhance discrimination between qualitatively different movement patterns, thereby reducing misrecognition and increasing the instructional reliability of the program during lessons.

In addition to improving recognition reliability, the feedback system was enhanced to support students' self-regulation during physical activity. While the initial version of the program provided visual feedback only, student participants suggested that additional feedback modalities would help them notice and correct incorrect movements more immediately. Based on this input, auditory feedback was incorporated alongside visual cues, allowing students to receive immediate and intuitive signals when their movement deviated from the expected form. Teachers reported that this multimodal feedback supported quicker correction and reduced the need for repeated verbal intervention during practice.

**Figure 2:**
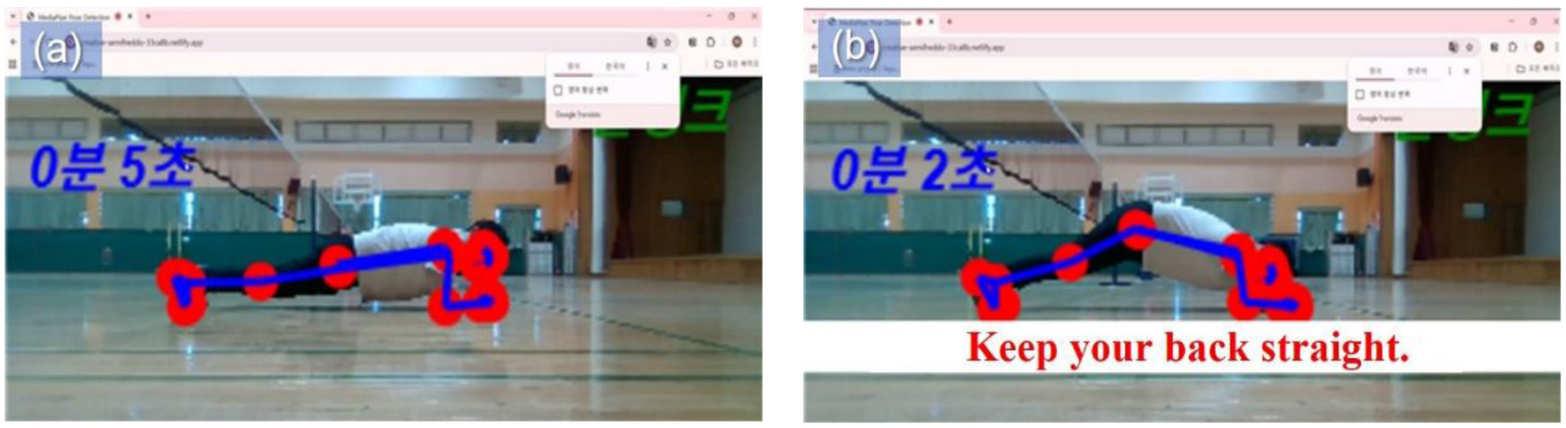
Motion recognition in the plank exercise AI training program. The image displays a single participant for privacy protection and interface clarity. The system was implemented during regular, full-class PE sessions in a typical school environment. **(a)** Maintaining a proper plank posture starts the timer **(b)** incorrect posture stops the timer and triggers feedback (“Keep your back straight”).

Furthermore, the program was adapted to accommodate exercise types that are evaluated based on duration rather than repetitions. Teachers noted that many fitness activities in school physical education, such as plank exercises, emphasize sustained posture rather than repeated movements. To reflect this pedagogical characteristic, a time-based tracking function was introduced for isometric exercises. As illustrated in Figure 2, students received immediate feedback when maintaining an appropriate posture and were alerted when posture deviations occurred, enabling them to adjust their performance in real time.

Through these iterative refinements, the program evolved to better support diverse instructional goals in physical education by improving recognition reliability, expanding feedback modalities, and accommodating different types of physical activities. These changes enhanced the program's pedagogical usefulness in classroom settings and supported both student engagement and teachers' capacity to provide timely, individualized feedback during lessons. For transparency, supplementary details regarding the refinement of motion recognition criteria are provided in Appendix B.

#### 3.1.3 Enhancing practicality and linking with exercise practice records

In this study, the program was implemented as a web-based system to enhance its practical utility in school settings. Unlike previous studies that primarily relied on locally executed environments or standalone applications (4, 5, 33, 34), this approach avoided common constraints such as device compatibility issues, operating system differences, complex installation procedures, and difficulties associated with updates. By eliminating the need for local installation, the program was designed to be readily usable within the time and resource constraints of physical education classes.

Students accessed the program through a standard web browser or by scanning a QR code, allowing immediate use during lessons without additional setup. When updates were required, they were applied centrally and reflected instantly in the latest version, enabling continuous use without maintenance on individual devices. This web-based design enhanced both accessibility and scalability, making the program suitable for repeated use in dynamic instructional environments such as physical education classes.

**Figure 3:**
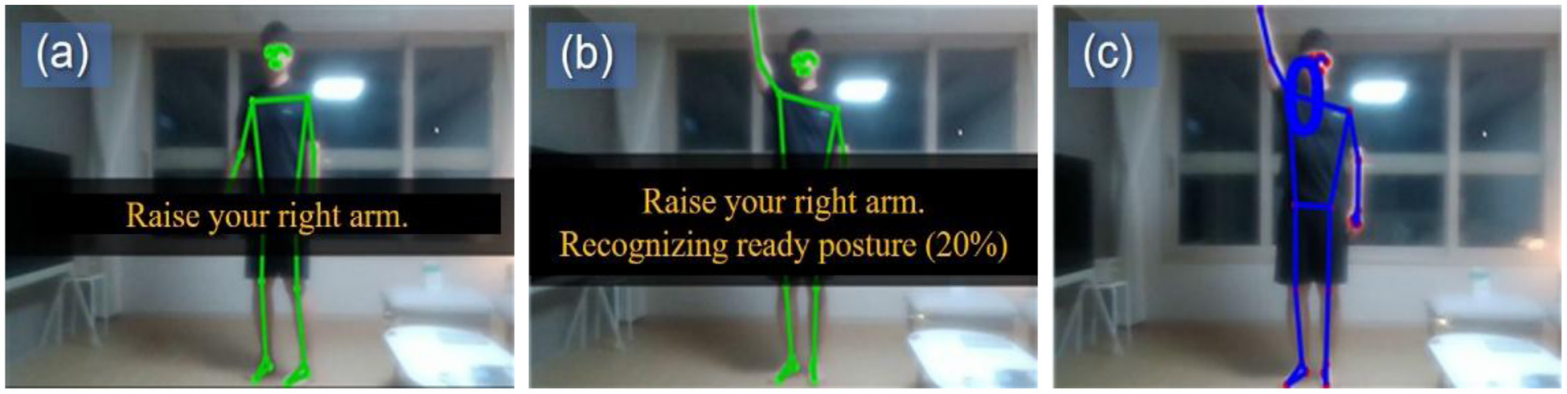
User interface flow for guiding the preparatory posture and activating motion recognition in the program. For privacy protection and clarity, the figure displays only the instructional interface; in practice, this material was used during full-class PE sessions in a typical school setting. **(a)** The system provides visual guidance for the ready posture while motion recognition is disabled. **(b)** Once the learner maintains the correct ready posture, the system begins recognizing it and displays a progress indicator until the posture is stabilized. **(c)** When the ready posture has been recognized with sufficient stability, the system confirms completion and activates real-time motion tracking for the exercise.

To further improve practical usability during lessons, a preparatory movement detection feature was introduced at the initial stage of program use. In conventional motion recognition systems, recognition often begins immediately upon program activation, which can lead to misrecognition when learners are adjusting devices or moving into position. To address this issue, the program was designed to activate full-scale motion recognition only after detecting a learner's ready posture. This design supported smoother transitions into exercise practice and reduced recognition errors caused by incidental movements. To illustrate this process of guiding learners into the preparatory posture and activating real-time motion recognition, we present in Figure 3 an example of the instructional material used in class.

To support the practical use of the program beyond single class sessions, an exercise logging function was implemented to enable the recording and sharing of students' exercise practice data. This function allowed teachers to receive records of students' exercise participation and performance, supporting ongoing monitoring and instructional decision-making. Technical details of the logging and data transmission system are provided in Appendix C.

Through these design choices, the program was developed to function not only as an in-class instructional tool but also as a means of extending physical education practice beyond individual lessons. By facilitating ease of access, minimizing technical barriers, and supporting the collection of practice records, the program enhanced its applicability for sustained use in school physical education contexts.

#### 3.1.4 Establishing a student-led program development environment

The AI program developed in this study was designed not merely as a classroom tool but as a participatory learning environment in which students could engage directly in the program development process and reflect on their own movement performance.

**Figure 4:**
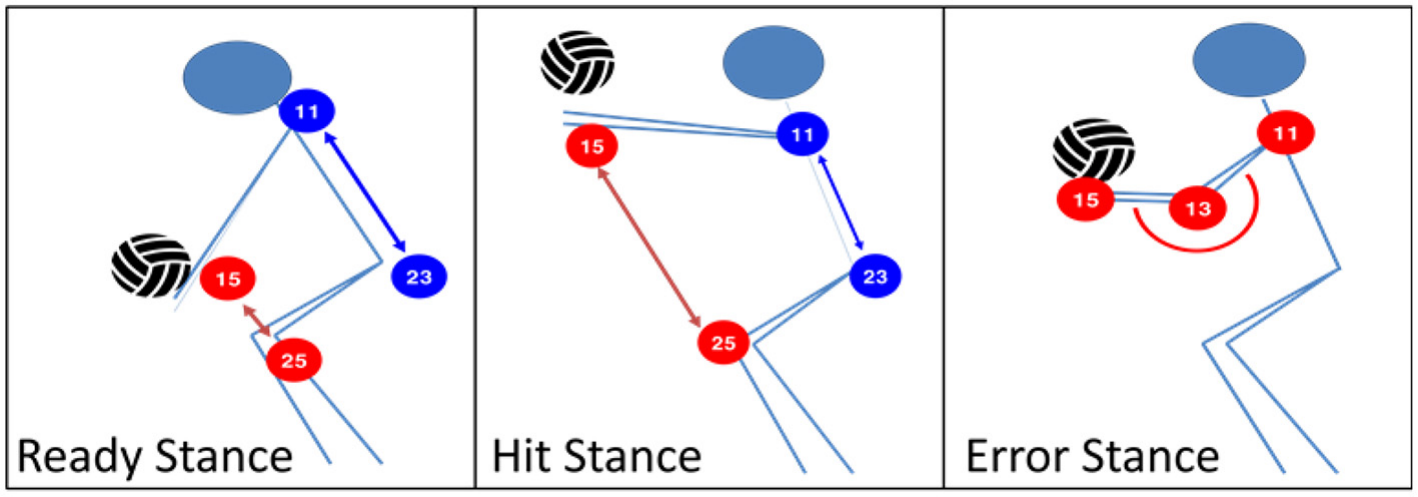
Examples of Students' Visual Representations of Volleyball Underhand Pass Movements.

First, as shown in Figure 4, students were guided to represent the target exercise by decomposing it into three key movement phases. This process supported students in analyzing the structure of the movement and identifying salient features related to posture, timing, and coordination. A notable learning episode was observed when students articulated these features by describing positional relationships among body parts and their changes across movement phases, thereby externalizing their kinematic understanding in a visual and explicit form.

In addition, the program provided a web-based environment that enabled systematic recording and feedback of learners' exercise participation through preparatory posture recognition and practice-record linkage functions. These features allowed teachers and students to review participation records and monitor changes across repeated sessions, supporting reflection on both performance and learning processes.

Building on this structure, the program supported iterative refinement by enabling teachers and students to collaboratively adjust feedback conditions and functional elements in response to emerging classroom needs. Through this process, students were positioned not as passive users of AI technology but as active contributors who engaged in analyzing, representing, and refining the instructional use of the program.

### 3.2 Educational implications of the AI program in PE

#### 3.2.1 AI-driven transformation of instruction: developing lesson-tailored tools and managing student growth through data

The program developed in this study enabled teachers not to remain mere end-users but to directly develop instructional tools for teaching and learning. Because the code used in this study has a simple structure, even teachers with limited expertise in coding can modify the program to recognize new exercise functions simply by editing a subset of joint-coordinate code. In this way, teachers can readily create programs that discriminate among movements in various events—not only basic fitness exercises but also ball-game skills such as volleyball and basketball—and provide appropriate feedback to students. This demonstrates an important outcome of the program, namely its potential to evolve into a lesson-tailored instructional tool for teachers.

In a badminton unit, one physical education teacher reported creating and using an AI program that provides feedback on the high-clear motion. By comparing the difference between the elbow and shoulder y-coordinates, the program detected the backswing, and by tracking changes in elbow angles, it distinguished between extending the elbow during the swing and the follow-through, operationalizing these three movements for students' daily repetitive practice. What the teacher valued most was the ability to design this tool to fit the specific needs of their own class (PE Teacher 5).

In addition, the program was integrated with Google Apps Script so that each student's exercise performance records could be automatically stored and managed. The log items included student ID, name, exercise duration, type of movement, and number of repetitions, enabling teachers to systematically monitor each student's level of exercise participation. Notably, the program can support not only repetitive movements but also a variety of isometric (static) training exercises such as planks.

This level of extensibility makes it possible to apply the program even to areas that have traditionally been difficult to assess in physical education, such as muscular endurance and balance, allowing teachers to evaluate and manage various components of students' physical fitness in a data-driven way. Beyond merely confirming achievement, these data-based records function as resources that can be used directly for lesson feedback and instructional design.

I applied the AI program in a health fitness class. Most of the students who participate have low fitness levels, so they dislike exercising in front of others. They said they would work out at home every day, but I had no way to verify it. So I gave them two links, a squat program and a plank program that I created, and told them to exercise at home. The students can work out at home while receiving AI feedback, and as the teacher I can receive their data, so it seems very useful (PE Teacher 5).

The teacher was able to identify how much and what kinds of exercise a particular student engaged in during class and in daily life, and on this basis could analyze the causes of the student's low fitness or plan supplementary instruction for specific motor skills. Conversely, when ongoing records showed an upward trend in exercise performance, learners gained a sense of achievement and teachers could provide positive feedback grounded in evidence. These outcomes enabled teachers to observe and manage each student's exercise engagement on a daily basis and made it possible to run physical education lessons in a data-driven manner. In addition, by making students' typically invisible exercise habits more observable and trackable over time, the system created a foundation for school-based health promotion efforts targeting students with low fitness and limited physical activity.

#### 3.2.2 Strengthening core learning in physical education: realizing AI-integrated education in PE

The student-led program development process created an environment in which students moved beyond being passive users of AI and engaged in analysing movement characteristics and designing feedback conditions for exercise performance. Through this process, students examined how different movement patterns could be distinguished based on positional relationships among body parts and applied this understanding to develop new exercise functions.

To design feedback for specific motor skills, students first needed to analyse the structure and key characteristics of each movement. Students reported experimenting with performing movements in various ways to identify common errors and discussing what types of feedback would be appropriate for supporting correct execution. As a result, students began to consider motor learning from the perspective of an instructor rather than solely as performers.

In this process, students demonstrated increased engagement with the core learning goals of physical education by articulating movement features, identifying errors, and refining feedback conditions. One student reflected on this experience as follows:

I was amazed that I could develop an AI program myself. It made me think about identifying the characteristics of each motor skill and how to teach them to the AI. Thinking of myself as the one doing the teaching led me to reflect much more deeply on each movement (Student 1).

Across multiple sessions, several students demonstrated similar shifts in perspective, reporting increased attention to movement structure, error patterns, and feedback design during program refinement. Across iterative cycles of program development and refinement, students repeatedly engaged in breaking down movements into key components, testing conditions for recognition, and adjusting feedback based on observed outcomes. These activities reflected a systematic and analytical approach to problem solving within the context of physical education learning.

Through these experiences, students not only deepened their understanding of physical activity but also engaged in integrative learning that connected movement analysis with structured problem-solving processes.

## 4 Discussion

### 4.1 Significance of the AI teaching–learning program developed and improved through participatory design

Although this study did not aim to quantify physiological health outcomes, it nevertheless demonstrates that the AI-supported system contributed to school health promotion by creating more opportunities for sustained physical activity, supporting the safe participation of low-fitness students, and enabling continuous monitoring of exercise engagement across both class-based and home-based contexts. By situating AI use within everyday physical education practice, the program addressed persistent challenges in school-based health promotion, particularly the difficulty of sustaining engagement and providing individualized support in heterogeneous PE classes.

Within this health-oriented educational context, the MediaPipe-based AI teaching–learning program developed in this study was iteratively refined through a participatory action research (PAR)-informed co-design process that incorporated teachers' and students' classroom-based feedback. In the initial push-up module, reliance on a single-joint reference (e.g., elbow position) resulted in frequent false positives, indicating that simplified recognition rules were insufficient to capture the complexity of authentic movement performance in PE lessons. This finding highlighted the importance of accounting for context-dependent movement variations in real classroom environments. In response, the recognition logic was extended to incorporate combinations of pose landmark coordinates (X–Y) and geometric relations such as distances and joint angles, aligning with recent efforts to enhance the precision and robustness of AI-based motion recognition in sport and exercise contexts (11, 29).

Beyond improvements in recognition accuracy, participatory refinements enhanced the pedagogical fit and scalability of the program. Counting and timer functions were introduced to accommodate both repetition-based exercises (e.g., squats and push-ups) and static postural tasks (e.g., planks), thereby enabling feedback to align with varied instructional goals and exercise modalities commonly used in school PE (5, 24). Importantly, the system was intentionally designed with a simplified rule structure so that, with basic guidance, teachers and students could adjust landmark-based criteria and threshold values to extend the program to additional movements without rebuilding the entire model. This design choice supported teacher agency and reduced dependence on external technical experts.

Finally, preparatory posture detection and web-based record linkage further enhanced the field applicability of the program by improving accessibility and usability in time-constrained lesson contexts. These features allowed the system to support not only immediate formative feedback during practice but also longer-term record keeping, thereby linking in-class activity with out-of-class exercise participation through lightweight spreadsheet-based integration (35). Overall, this study illustrates how an AI tool can evolve beyond executing a developer's initial design and become a flexible, classroom-responsive system shaped by situated pedagogical demands, with clear relevance for sustainable, school-based health promotion.

From the perspective of technological and pedagogical agency, our findings suggest that the expert-defined MediaPipe backbone (technological agency) provided a stable basis for extracting pose information, while the PAR process enabled teachers and students to exercise pedagogical agency by redefining how this information was interpreted and used for health-oriented feedback and lesson design. In other words, the bottom-up and student-centered character of the intervention emerged not from altering the pose-estimation model itself, but from co-configuring the rule sets, feedback messages, and classroom routines built on top of it. This illustrates how technically expert-driven AI components can be integrated into PE in ways that support, rather than undermine, teacher and student agency.

### 4.2 Presenting the feasibility of AI-integrated education in physical education classes

The second key finding suggests that a participatory AI development process can meaningfully shift the roles of both learners and teachers in physical education, thereby illustrating the feasibility of AI-integrated education in authentic PE settings. From a school-based health and education perspective, this feasibility lies not only in the technical operation of AI systems but also in their capacity to be embedded within everyday instructional practice in ways that are inclusive, scalable, and pedagogically sustainable. Rather than positioning AI merely as a “tool” to improve learning outcomes, the findings indicate that the development-and-testing environment itself can function as a productive learning space in which learners and teachers engage in ongoing inquiry, refinement, and reflection.

First, as students “taught” exercise movements to AI, they analyzed key movement principles, translated these into rule-based criteria such as landmark relations and thresholds, and iteratively tested and revised their designs. Through this process, students shifted from passive consumers of knowledge to active producers who created concrete artifacts, including recognition rules and feedback logic, consistent with constructionist perspectives that emphasize learning through making (36). Importantly, students were not only performing the movements but also thinking about and representing them, suggesting that physical education can support integrative learning that connects health-related movement understanding with digital literacy and computational thinking (37, 38).

Second, teachers moved beyond being end users of ready-made technology to acting as curriculum designers who evaluated misrecognition cases, adjusted feedback criteria, and adapted the system to local instructional aims and classroom constraints. This shift indicates that AI functioned as a pedagogical partner that extended teachers' professional agency and supported context-sensitive decision making, rather than prescribing standardized forms of instruction. At the system level, the integrated structure comprising web-based delivery, preparatory movement recognition, and exercise record linkage supported systematic feedback and monitoring of learners' exercise participation. Such features point toward the possibility of more sustainable, data-informed instructional practices that can be maintained over time within school physical education settings (5).

Overall, the study highlights the practical feasibility of AI-integrated physical education by demonstrating how AI can mediate a classroom-responsive learning ecosystem in which both students and teachers learn through iterative design and refinement. Taken together, these outcomes illustrate a feasible pathway for implementing AI-integrated education in physical education that not only enhances learning processes but also extends the pedagogical and health-related functions of the PE curriculum beyond physical performance alone.

### 4.3 Research implications

The findings of this study offer three scholarly implications for AI-integrated education in physical education (PE). First, the study demonstrates that participatory design, specifically PAR-informed co-design, can serve as a rigorous approach for aligning technological advancement with pedagogical practice. Rather than treating classroom misrecognition as a marginal technical flaw, iterative cycles of problem identification, rule adjustment such as landmark relations and threshold values, and re-testing enabled the tool to achieve pedagogical fit in authentic PE contexts. This suggests that the evaluation of AI tools in educational settings should consider not only measures of algorithmic accuracy but also how design decisions are negotiated, adapted, and stabilized through situated classroom use.

Second, the study contributes design-oriented insights by illustrating a rule-based and adaptable architecture for pose estimation feedback systems. The combination of simplified landmark-based criteria, exercise-type-sensitive logic including counting and timer functions, and lightweight web-based spreadsheet record linkage provides a set of actionable design principles that can inform future development of scalable AI-supported PE systems. Importantly, such an architecture lowers the barrier to contextual adaptation by teachers and supports sustained use beyond single lessons or short-term interventions.

Third, the study extends discussions of integrative learning by empirically illustrating how digital literacy and computational thinking can be meaningfully embedded within PE content through the act of teaching movements to AI. This reframes AI integration from an add-on tool aimed solely at enhancing learning outcomes to a learning environment that enables both learners and teachers to engage in inquiry, representation, and iterative refinement. Taken together, these implications highlight the value of co-designed AI systems as a pathway toward sustainable and learner-centered feedback practices in physical education and related educational contexts.

From a public health perspective, the capacity to monitor and support students' physical activity participation across both lesson-based and home-based contexts suggests that AI-supported PE systems can contribute to school-based strategies for promoting youth health and physical activity. Moreover, by framing AI as a means to support safe, inclusive, and health-oriented participation in school physical education rather than as an end in itself, this study aligns with international recommendations that emphasize human-centered and equity-focused applications of AI in education (39).

### 4.4 Research limitations and suggestions for future research

This study was conducted as a qualitative case study with a small number of participants to enable in-depth exploration. Accordingly, the findings should be interpreted in terms of transferability to similar school-based contexts rather than statistical generalizability across all educational settings. In addition, because the researcher actively engaged in the research process as an insider with an established trust relationship with participants, the researcher's positionality may have influenced data generation and interpretation. Although multiple strategies were employed to enhance trustworthiness, including iterative member checking, peer debriefing, and the maintenance of an audit trail where applicable, some degree of interpretive influence cannot be entirely ruled out. The study may also be subject to contextual and sampling-related constraints, such as participants' prior interest or experience in digital tools, which could have shaped engagement with the participatory development process.

From a technical perspective, webcam-based two-dimensional motion recognition can produce errors depending on camera angle, lighting conditions, and body occlusion, and is unlikely to match the precision of three-dimensional motion capture systems (40). While this study did not conduct formal benchmarking of pose-estimation accuracy using precision, recall, or related metrics, such evaluations were beyond the scope of the present implementation-focused research and should be addressed in future work. Furthermore, as the program incorporates web-based logging and data transmission features, additional work is required to strengthen data governance, including privacy protection, access control, and secure data storage, particularly when considering large-scale or long-term implementation beyond the study context.

Based on these limitations, several directions for future research are proposed. First, mixed-methods or quasi-experimental studies conducted over extended periods across multiple schools and more diverse student populations would help to examine the effectiveness and sustainability of AI-integrated PE programs, including outcomes related to physical competence, physical activity behavior, and digital or computational competencies. Second, establishing and examining online teacher community platforms for sharing, collaboratively refining, and ethically managing teacher-developed AI tools could provide insights into how teacher professional development, pedagogical agency, and sustainable implementation practices evolve within such ecosystems. Finally, future research could extend the participatory AI development model to other subject areas, such as music, art, or science experiments, and investigate the conditions under which cross-curricular transfer of AI-integrated pedagogical practices is both feasible and educationally meaningful.

## 5 Conclusion

This study examined the development process and educational significance of an AI-based physical education teaching–learning model through an integrated approach combining participatory action research (PAR) and rapid prototyping instructional system design (RPISD). The findings demonstrate that a participatory development process using accessible, web-based MediaPipe technology can effectively address the limitations of traditional top-down, expert-driven research models by closely integrating classroom-based needs with technical implementation.

Beyond technical development, the participatory process fostered meaningful educational growth among both students and teachers. By teaching exercise movements to AI, students moved from passive consumers of knowledge to active producers who analyzed movement principles and created their own instructional tools. Teachers likewise expanded their professional roles by shifting from technology users to curriculum designers who reinterpreted digital tools in alignment with their instructional philosophies. These findings suggest that physical education can extend beyond physical activity to support integrative learning experiences that include digital literacy and computational thinking.

Most importantly, this study contributes not only an AI-supported instructional tool but also a concrete methodological pathway for participatory AI-integrated education, in which teachers and students collaboratively shape technology to address contextual educational challenges. From a public health perspective, this approach illustrates how AI-integrated physical education can function as a school-based strategy for supporting inclusive participation, sustained physical activity, and data-informed monitoring of students' exercise engagement. By foregrounding human agency and contextual relevance, the study offers a shift from technology-driven solutions toward educational practices that support meaningful, equitable, and health-oriented uses of AI in physical education and related learning environments.

## Data Availability

The raw data supporting the conclusions of this article will be made available by the authors, without undue reservation.
