# Supplementary material for: Development and implementation of a MediaPipe-based AI teaching-learning model in school physical education for health promotion
Source: Front Public Health. 2026 Mar 20;14:1786427. doi: 10.3389/fpubh.2026.1786427 (PMC13048069; doi:10.3389/fpubh.2026.1786427)
Supplement: Supplementary file 1 [file Data_Sheet_1.pdf]

Supplementary Material

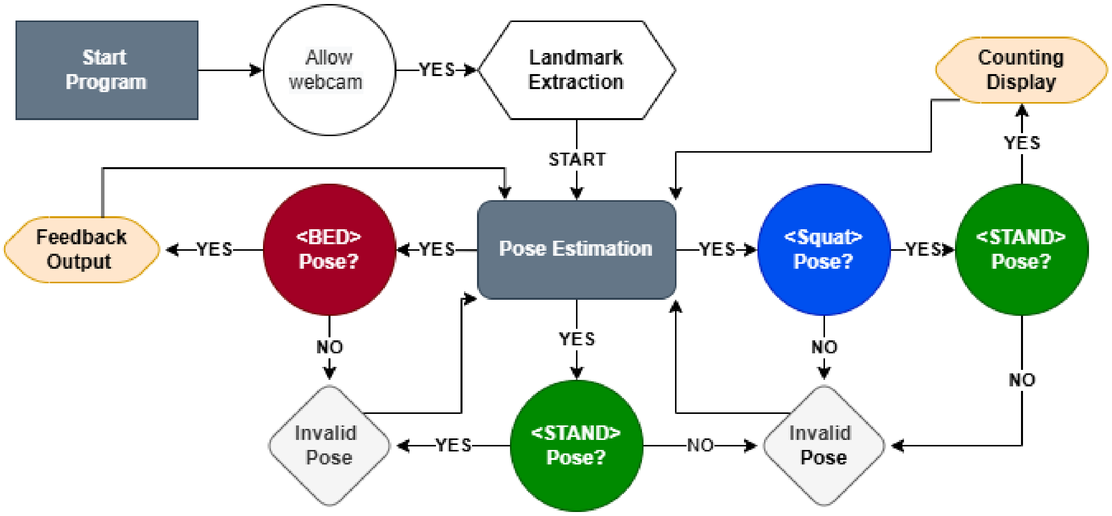

Appendix A: Operational Structure of the MediaPipe-based AI teaching-learning Program

Appendix B: Improvements in Program Recognition Accuracy through Code Enhancements

Category	Existing algorithm	Improved algorithm
Decision criterion	Judges the degree of upper-body flexion by calculating the elbow–shoulder–wrist angle (angL1)	Determines whether actual exercise is being performed by additionally comparing the Y-coordinate difference between the shoulder (11) and heel (29)
Core code	else if (angL1<200)	else if ((angL1 < 200 && ((points[11].y - points[29].y) < 0.4

Appendix C: Configuration and roles of the logging and transmission system for the exercise-record linkage function.

Category	Key technology	Role	Detailed description
User Interface	HTML, CSS, JavaScript	Requesting data transmission	- When “Send Record” is tapped, the submitRecord() function is called to initiate the data transmission process.
Data transfer	Fetch API (POST)	Data exchange between client and server	- Converts the exercise log data into JSON format and sends an asynchronous request to the Google Apps Script web app URL.
Data processing	Google Apps Script	Receiving and processing data & recording data in Google Sheets	- The script deployed as a web app receives POST requests through the doPost(e) function.<br>- It parses the transmitted JSON data to extract information such as student ID, name, and number of repetitions.<br>- Using the SpreadsheetApp service, it adds the data as a new row to the specified sheet.
Database	Google Sheets	Storing and managing exercise log data	- Serves as a database in which exercise records transmitted from users are cumulatively stored.
